# Heterogeneity Analysis of Bladder Cancer Based on DNA Methylation Molecular Profiling

**DOI:** 10.3389/fonc.2022.915542

**Published:** 2022-06-07

**Authors:** Shuyu Wang, Dali Xu, Bo Gao, Shuhan Yan, Yiwei Sun, Xinxing Tang, Yanjia Jiao, Shan Huang, Shumei Zhang

**Affiliations:** ^1^College of Information and Computer Engineering, Northeast Forestry University, Harbin, China; ^2^Department of Radiology, The Second Affiliated Hospital of Harbin Medical University, Harbin, China; ^3^Department of Neurology, The Second Affiliated Hospital of Harbin Medical University, Harbin, China

**Keywords:** bladder cancer, DNA methylation, molecular subtypes, subtype specific biomarkers, heterogeneity analysis

## Abstract

Bladder cancer is a highly complex and heterogeneous malignancy. Tumor heterogeneity is a barrier to effective diagnosis and treatment of bladder cancer. Human carcinogenesis is closely related to abnormal gene expression, and DNA methylation is an important regulatory factor of gene expression. Therefore, it is of great significance for bladder cancer research to characterize tumor heterogeneity by integrating genetic and epigenetic characteristics. This study explored specific molecular subtypes based on DNA methylation status and identified subtype-specific characteristics using patient samples from the TCGA database with DNA methylation and gene expression were measured simultaneously. The results were validated using an independent cohort from GEO database. Four DNA methylation molecular subtypes of bladder cancer were obtained with different prognostic states. In addition, subtype-specific DNA methylation markers were identified using an information entropy-based algorithm to represent the unique molecular characteristics of the subtype and verified in the test set. The results of this study can provide an important reference for clinicians to make treatment decisions.

## Introduction

In recent years, cancer has become an important killer of human health, and seriously threatens people’s life and health. It is generally believed that cancer is caused by the accumulation of mutations in cancer susceptibility genes and resulting abnormal cell growth, but a large number of recent studies have shown that in addition to genetic variation, abnormal DNA methylation also plays an important role in the occurrence and development of cancer (1). DNA methylation is the most extensively documented epigenetic modification that can influence cell fate and gene expression (2, 3), which finally leads to the inhibition of gene expression through formation of heterochromatin in the gene regulatory region (4).

There are many studies have demonstrated the importance of DNA methylation (5–9). Numerous studies have shown that global hypomethylation of DNA and hypermethylation of cytosine-phosphate-guanine (CpG)-enriched regions are common in cancers (10–13). Methylation of promoters inhibits gene transcription, and abnormal methylation is one of the main causes of genomic instability, oncogene activation and tumor suppressor gene suppression. For example, abnormal methylation in colorectal tumors is characterized by hypermethylation in promoters and transcriptional silencing of tumor suppressors or DNA repair genes (14–17), coexisting with global methylation loss that leads to chromosomal and microsatellite instability and oncogene activation (18). Both promoter hypermethylation and global hypomethylation are markers of the early stage of colorectal cancer (19–22). Therefore, abnormal methylation may contribute greatly to the pathogenesis and progression of cancer. In addition, there are many studies of disease based on computational methods (23–25).

Bladder cancer is one of the most common malignant tumors in urology, and its incidence is increasing year by year. About 70% of newly discovered bladder cancer is non-invasive bladder cancer, but nearly 70% of patients relapse after surgical resection of the primary tumor, and 30% of them progress to invasive bladder cancer. Invasive bladder cancer has a poor prognosis and is the main cause of eventual metastasis and death of bladder cancer patients (26). Bladder cancer can be divided into two categories according to the invasion degree of tumor and whether it invades muscle layer. Nearly 70% of these cancers are non-muscle invasive bladder cancer (NMIBC). The main treatment methods for NMIBC are transurethral resection of the bladder tumor and local perfusion therapy of bladder (TURBT). TURBT is a minimally invasive surgery with little trauma and fast recovery. Patients have a relatively good prognosis (27). About 20% ~ 30% are muscle-infiltrating bladder cancer, which is prone to recurrence and distant metastasis after operation due to its high degree of malignancy and complicated treatment. Therefore, early identification of cancer types in patients with bladder cancer is of great significance for cancer treatment.

A large number of studies have focused on abnormalities in DNA methylation and its important role in the occurrence and development of bladder cancer. Kawakami et al. reported for the first time that MSH3 epigenetic regulation by means of DNA methylation might contribute to gene silencing, being implicated in bladder cancer carcinogenesis (28). In addition, there are many researchers aimed of the prognosis of bladder cancer at the level of DNA methylation. Recently, with BLCA sample transcriptome data and methylation data from The Cancer Genome Atlas (TCGA), 18 target genes were identified and the signature based on them was considered an effective and independent prognostic factor (29). However, the existence of tumor heterogeneity leads to the inconsistency of tumor phenotype, and the efficacy and prognosis of different patients are also significantly different. These differences not only pose great challenges to the clinical treatment of bladder cancer, but also reflect the importance of precision medicine. Genetic variation is the core of tumor heterogeneity. There is a wide range of genetic diversity in tumors, and genomic instability leads to a large number of mutations, which is the main cause of genetic heterogeneity in tumors (30). But epigenetic changes, including DNA methylation, also play an important role in cancer development and perhaps in the molecular heterogeneity of cancer. A previous study showed that BRCA1 promoter methylation was correlated with clinical breast cancer stages (31). Thus, DNA methylation status may be used as a marker for cancer molecular subtyping.

In fact, a large number of studies have been devoted to the analysis of molecular subtypes and DNA methylation heterogeneity of bladder cancer. Attempts have been made to unravel the complexity and refine these molecular subtypes based on biomarkers and pathways, mutations and copy number aberrations, or protein abundance (32). Lindskrog et al. performed an integrative multi-omics analysis of patients diagnosed with NMIBC and identified four classes reflecting tumor biology and disease aggressiveness (33). A comprehensive analysis of 412 muscle-invasive bladder cancers characterized by multiple TCGA analytical platforms, clustering by mRNA, lncRNA, and miRNA expression converged to identify subsets with differential epithelial-mesenchymal transition status, carcinoma-*in-situ* scores, histologic features, and survival (34). Recently, Ye et al. used DNA methylation to predict tumor molecular subtypes and efficacy of immunotherapy in bladder cancer (35). One previous study used DNA methylation profiling of bladder cancer samples obtained from the Illumina GoldenGate Methylation Bead Array and unsupervised clustering of those loci with the greatest change in methylation between tumor and non-diseased tissue was performed to defined molecular subgroups of bladder cancer (36). However, most of these analyses did not integrate DNA methylation and gene expression into a detailed classification of bladder cancer at the molecular level, nor did they provide specific biomarkers for individual molecular subtypes.

In this study, we addressed bladder tumor classification based on DNA methylation profiles of BLCA from The Cancer Genome Atlas (TCGA) database. The classification characteristics were obtained by integrating gene expression and DNA methylation data, then the molecular subtypes of bladder cancer were identified based on consistent clustering, and specific prognostic differences among these subgroups were analyzed. This classification system may help find new bladder cancer markers or molecular subtypes and more accurately subdivide bladder cancer patients. Additionally, our criteria will provide more targets for bladder cancer precision medicine by finding specific molecular markers for each subtype. Finally, the new molecular subtypes and subtype-specific molecular markers identified in this study were validated in an independent cohort from GEO database.

## Materials and Methods

### Data Acquisition and Processing

The Illumina Infinium HumanMethylation450 Bead Chip DNA methylation profile data and RNA-seq data of bladder cancer patients as well as clinical information and survival data of the samples were obtained from TCGA database (37), including 408 tumor samples, 14 normal control samples. The expression data were processed as follows: zero-valued entries were replaced by the minimal positive value of the dataset; the expression values were logarithmically transformed (base 2) to normalize the data. The methylation level of each probe was represented by β-value, which ranges from 0 to 1, corresponding to unmethylated and fully methylated, respectively. Probes with missing data in more than 70% of the samples were removed. The remaining probes with not available (NAs) were imputed using the k-nearest neighbors (KNN) imputation procedure. Unstable genomic sites, including CpGs in sex chromosomes and single nucleotide polymorphisms were removed. Because DNA methylation in promoter regions strongly influences gene expression (38), we selected CpGs within promotor regions. Promoter regions were defined as 2 kb upstream to 0.5 kb downstream from transcription start sites.

### Identification of Differentially Expressed Genes and Differentially Methylated CpG Sites

In this study, the differences of gene expression and DNA methylation were combined to classify patients, so the data sets were first used to screen differentially expressed genes and differentially methylated CpG sites between cancer samples and adjacent control samples of bladder cancer.

Differentially expressed genes were screened by samr R package. Genes that meet the following two conditions are identified as differentially expressed genes: foldchange > 2, q < 1. Differential methylated CpG sites were screened by minfi R package. The CpGs whose adjusted p value were less than 0.05 and the difference of the average β values were more than 20 percent were considered differentially methylated CpGs between cancer patients and adjacent control tissues. The Differentially expressed genes and differentially methylated CpG sites were displayed using heat maps, which were completed using heatmap.2 function. All processes were programmed using R software.

### Correlation Analysis of Gene Expression and DNA Methylation

Since hypermethylation in the gene promoter regions usually inhibits the expression of downstream genes, the methylation level of the gene promoter regions should be negatively correlated with the expression level of corresponding gene, that is, the higher the methylation level, the lower the corresponding gene expression level. Therefore, Pearson Correlation Coefficients between the differentially methylated CpG sites within promoter regions and differentially expressed genes were calculated, and CpGs whose DNA methylation levels significant negatively correlated (Pearson Correlation Coefficient less than 0, p < 0.05) with the corresponding gene expression levels were selected as classification characteristics, these CpG sites are the regulators of gene expression. Pearson Correlation Coefficient is calculated as follows:


(1) $$\text{r}=\frac{1}{n-1}\sum_{i=1}^{n}\left(\frac{{\text{X}}_{\text{i}}-\bar{\text{X}}}{\delta_{\text{X}}}\right)\left(\frac{{\text{Y}}_{\text{i}}-\bar{\text{Y}}}{\delta_{\text{Y}}}\right)$$

### Molecular Subtypes of BLCA Were Obtained by Consistent Clustering

Consensus clustering was performed using the ConcensusClusterPlus package (39) to determine subgroups of BLCA based on the characteristic CpG sites obtained in the previous step. The algorithm began by subsampling a proportion of items and features from the data matrix, where each subsample was partitioned into up to k groups by a user-specified clustering algorithm such as k-means, hierarchical clustering or a custom algorithm. This process was repeated for a user-specified number of repetitions, providing a method of representing the consensus across multiple runs of the clustering algorithm and assessing the stability of the discovered clusters. Pairwise consensus values, defined as ‘the proportion of clustering runs in which two items are grouped together’, were calculated and stored in a consensus matrix for each k. Then, for each k, a final agglomerative hierarchical consensus clustering using distance of 1-consensus value was completed and pruned to k groups, which were called consensus clusters. This algorithm determined “consensus” clusters by measuring the stability of clustering results from the application of a given clustering method to random subsets of data. In each iteration, 80% of the tumors were sampled, and the k-means algorithm, with the Euclidean squared distance metric, i.e.


(2) $$\text{d}={\sum_{\text{k}=1}^{N}{\left(x_{11}-y_{11}\right)}^{2}+\cdots +\left(x_{1k}-y_{1k}\right)}^{2}+\cdots +{\left(x_{1N}-y_{1N}\right)}^{2}$$

was used with k = 2 to k = 10 groups; these results were compiled over 100 iterations. After executing ConsensusClusterPlus, the cluster-consensus and item-consensus results were obtained. The graphical output results included heatmaps of the consensus matrices, which displayed the clustering results, consensus cumulative distribution function (CDF) plot and delta area plot, which allow us to determine an approximate number of clusters. The criteria to determine the number of clusters we considered were that the consistency within the clusters was relatively high, the coefficient of variation was relatively low and that there was no appreciable increase in the area under the CDF curve. The coefficient of variation was calculated according to the following formula:


(3) $$CV=\left(\frac{SD}{MN}\right)\times 100\%$$

in which SD represents the standard deviation, and MN represents the average of samples.

### Differential Prognostic Analysis of Molecular Subtypes

In order to test the differences except DNA methylation levels among the bladder cancer subgroups obtained, survival analysis was performed on the patients in these subgroups. Kaplan–Meier plots were used to illustrate overall survival among BLCA subgroups defined by DNA methylation profiles. The log-rank test was used to evaluate the significance difference among the clusters, p < 0.05 was considered significant. Survival analyses were performed using the survival package in R.

### Identification of Subgroup Specific DNA Methylation Biomarkers in Bladder Cancer

In this analysis, a quantitative approach for quantitative differentially methylated regions (QDMRs), which quantify methylation differences and identify DMRs from genome-wide methylation profiles by adapting Shannon entropy (40), was used to find the specific DNA methylation CpGs that were specifically hypermethylated or hypomethylated within particular bladder tumor subgroup. The quantification of DNA methylation difference across large numbers of samples and the identification of sample specificity plays important roles in genomic functional analyses. DMRs with different methylation statuses among multiple samples were regarded as possible epigenetic functional regions involved in transcriptional regulation. Thus, the identification of DMRs among multiple samples provided a more comprehensive survey for this study. With the rapid development of high-throughput detection technology, there have been considerable efforts in identifying DMRs from methylation profiles. However, the development of DNA methylation measurements proposed significant challenges for concurrent DMR methods. Shannon entropy, a quantitative measure of differences and uncertainty in data sets, has been widely applied in quantitative biology, such as identifying potential drug targets and tissue-specific genes. To quantify methylation differences and further identify DMRs across multiple samples, Zhang et al. adapted the Shannon entropy model and developed an improved approach, termed quantitative differentially methylated region (QDMR). QDMR was an effective tool for quantifying methylation differences and identifying DMRs across multiple samples. This approach can give a reasonable quantitative measure of methylation differences across multiple samples as well. We used the threshold that was determined by QDMR from the methylation probability model. Furthermore, QDMR can also measure the sample specificity of each DMR. For each DMR *r*, the entropy *H*_Q_ represents the methylation difference across all samples. For each sample *S*, the entrophy is $H_{Q/\bar{S}}$ the difference across samples that do not include sample *S*. Thus, the contribution of sample *S* to the whole methylation difference can be reflected by the entropy difference as:


(4) $$\Delta H_{r/S}=H_{Q/\bar{S}}-H_{Q}$$

And the categorical sample-specificity *CS_r/s_* can be defined as:


(5) $$CS_{r\;/S}=\left\{\begin{array}{l} \Delta H_{\;r/S}\times \;sign_{\;r/S},\Delta H_{\;r/S}>\;0\\ 0,\;\Delta H_{\;r/S}\;\leq \;0 \end{array}\right\}$$

where *sign_r,s_* is the sign of the difference between methylation level *m_r/s_* in sample *S* and the median methylation level of vector *m_r_* in region *r*, as described by Zhang et al. (40). Thus, the subgroup with the maximal absolute of the categorical sample-specificity *CS_r/s_* was determined as the specific subgroup corresponding to the particular CpG site.

### Functional Enrichment Analysis of Genes Corresponding to Specific CpGs

In this study, using DAVID (41, 42), a database used for annotation, visualization and integration of discoveries, we conducted a GO (Gene Ontology) biological functions enrichment analysis and a KEGG (Kyoto Encyclopedia of Genes and Genomes) pathways enrichment analysis towards the list of genes corresponding to specific CpGs, with p controlled within 0.05, which could find out the biological characteristics involved by our specific CpGs.

### Construction and Verification of Classification Model

In order to verify the accuracy of classification characteristics, robustness of subtypes and accuracy of subtype-specific CpG sites of the bladder cancer DNA methylation subtypes classified in this study, another set of external test data set (GSE52955) was retrieved from GEO database. The classification features (characteristics CpG sites) were used as the features of the model, TCGA data used in this study was used as the training set to build a SVM classifier model, and the accuracy of the model was verified by the ten-fold cross-validation method. The external test set data is then entered into the built model, which is used to classify the new samples.

## Results

### Acquisition of Classification Features

**Figure 1:**
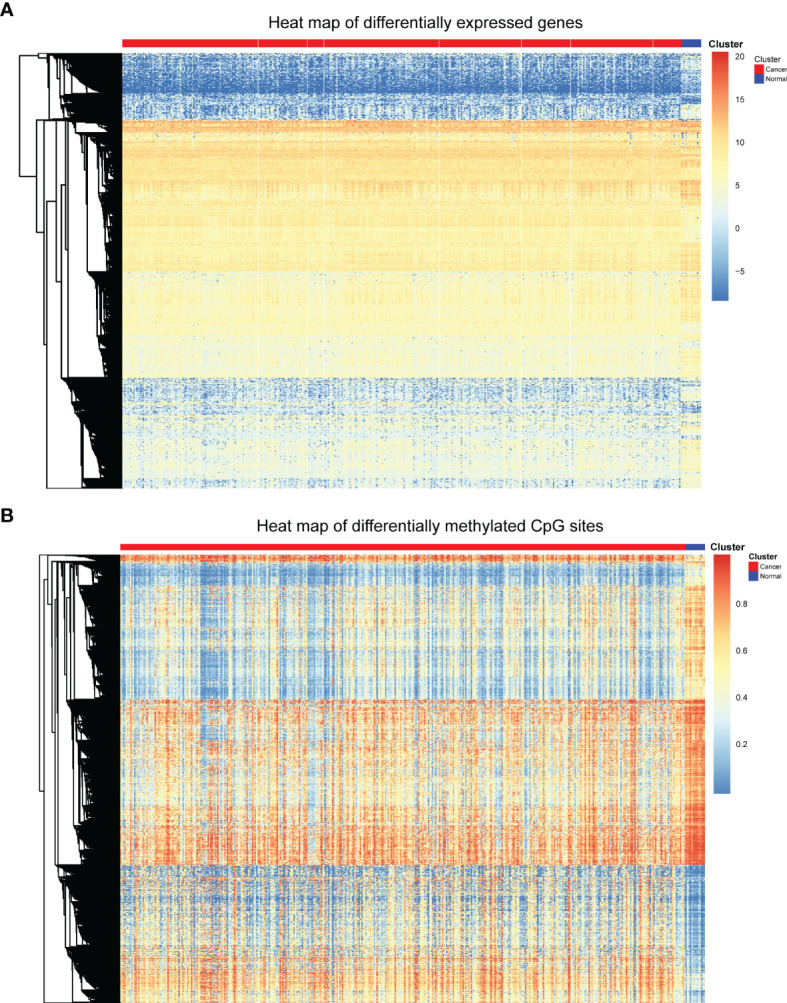
Heat map of differentially expressed genes and differentially methylated CpGs. **(A)** Heat map of differentially expressed genes. **(B)** Heat map of differential methylated CpG sites.

To obtain characteristics for molecular subtype classification, we first identify genes and CpG sites that differ between cancer and normal samples which are associated with cancer development. First, samr R package was used to screen differentially expressed genes as described above. 408 differentially expressed genes were obtained, and these differentially expressed genes were displayed by heat map. In the heat map, genes were represented by rows and patients were represented by columns, the red bars represent cancer patients, the blue bars represent normal tissue samples adjacent cancer samples, and the middle area were gene expression levels (**Figure 1A**). As can be seen from the heat map, these differentially expressed genes can clearly separate the bladder cancer patient samples from the para-cancer control samples.

Next, minfi R package was used to screen differentially methylated CpG sites. Through the processes mentioned above, 9702 differentially methylated CpGs between bladder cancer patients and control samples were identified. The differentially methylated CpGs were also shown in the heat map (**Figure 1B**). The heat map displays the methylation levels of differentially methylated CpGs in cancer samples and adjacent control samples. The rows represent CpG sites, the columns represent patients, and the colors represent the levels of DNA methylation. As can be seen from the heat map, these differentially methylated CpG sites can also clearly separate the bladder cancer patient samples from the para-cancer control samples.

Since the methylation level of gene promoter region was negatively correlated with the expression level of corresponding gene, the CpG loci which significant negative correlation with gene expression were extracted (Pearson Correlation Coefficient less than 0, p < 0.05). Finally, 986 CpG loci were obtained and analyzed as the classification features.

### Different Molecular Subtypes of Bladder Cancer Were Obtained Based on Consistent Clustering Algorithm

**Figure 2:**
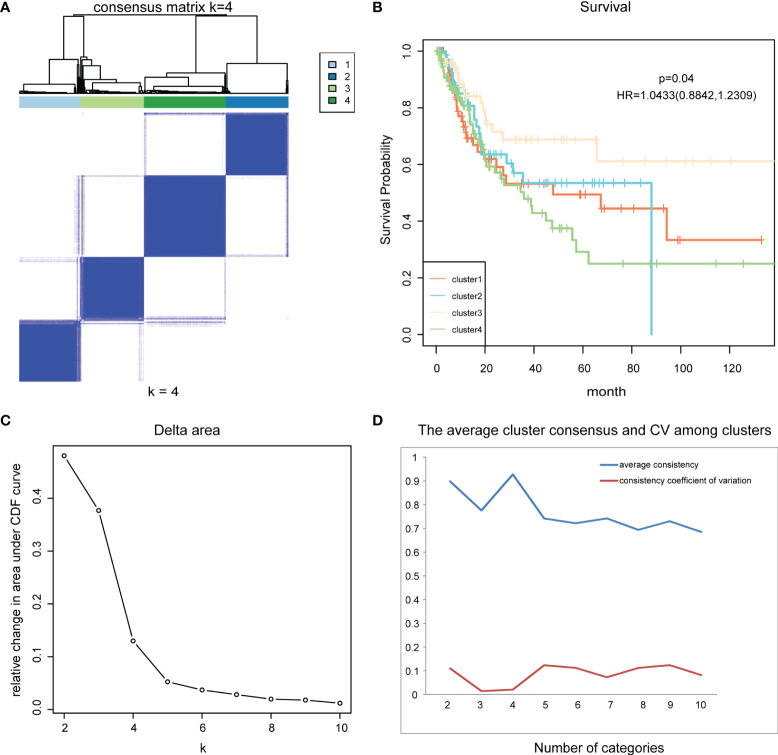
Consensus clustering and survival analysis. **(A)** The color-coded heatmap corresponding to the consensus matrix for k = 4. **(B)** The survival curves of four DNA methylation subtypes of bladder cancer. **(C)** Delta area curve of consensus clustering. **(D)** The average cluster consensus and coefficient of variation among clusters for each category number k.

Next, consensus clustering based on the β values of the 986 CpG sites obtained was performed to obtain distinct DNA methylation molecular subtypes of bladder cancer. To determine the appropriate number of subgroups, the average cluster consensus and the coefficient of variation among clusters were calculated for each category number. In this study, the cluster number selection criteria we considered were relatively high average consistency within the clusters, relatively low coefficient of variation, and maximum area change under the CDF curve. The consensus matrix was naturally a better visualization tool to help assess the clusters’ composition and number. We associated a color gradient from 0–1, with white corresponding to 0 and dark blue corresponding to 1, and assume the matrix is arranged so that items belonging to the same cluster are adjacent to each other. In this arrangement, a matrix corresponding to a perfect consensus will show a color-coded heatmap characterized by blue blocks along the diagonal on a white background. The color-coded heatmap corresponding to the consensus matrix obtained by applying consensus clustering to these cases is shown in **Figure 2A**, and represents the consensus for k = 4, which displays a well-defined 4-block structure. It has the largest area change under CDF curve, the highest average consistency within the class, and the lowest consistency coefficient of variation (**Figures 2C, D**). Therefore, we determine the appropriate number of categories as 4. So, all bladder cancer patients were divided into four DNA methylation molecular subtypes.

### Prognostic Analysis of Different Molecular Subtypes

After consistent clustering was used to identify DNA methylation subgroups in bladder cancer, we then examined whether there were differences among the subgroups in addition to DNA methylation levels. We examined the differences in prognosis among the four DNA methylation subgroups.

Kaplan-Meier survival analysis was performed for these four subtypes using functions survfit() and survdiff () in R package “Survival”, and log rank test was used to determine the statistical significance of survival differences. Results showed significant prognostic differences among the four subgroups (p = 0.04) (**Figure 2B**). This indicates that there are significant differences in the prognostic status of patients among the four DNA methylation molecular subtypes of bladder cancer, which can provide an important reference for clinicians to predict the survival status of patients and timely change the treatment plan.

### Identification and Analysis of DNA Methylation Biomarkers Specific to DNA Methylation Subtypes

After identifying the DNA methylation molecular subtypes of bladder cancer using unsupervised consistent clustering, the present study focused on DNA methylation markers specific to each subtype. These markers could represent the unique characteristics of each subtype, and their screening can provide a basis for the diagnosis of DNA methylation subtypes, and facilitate the better translation of research results into clinical application.

**Figure 3:**
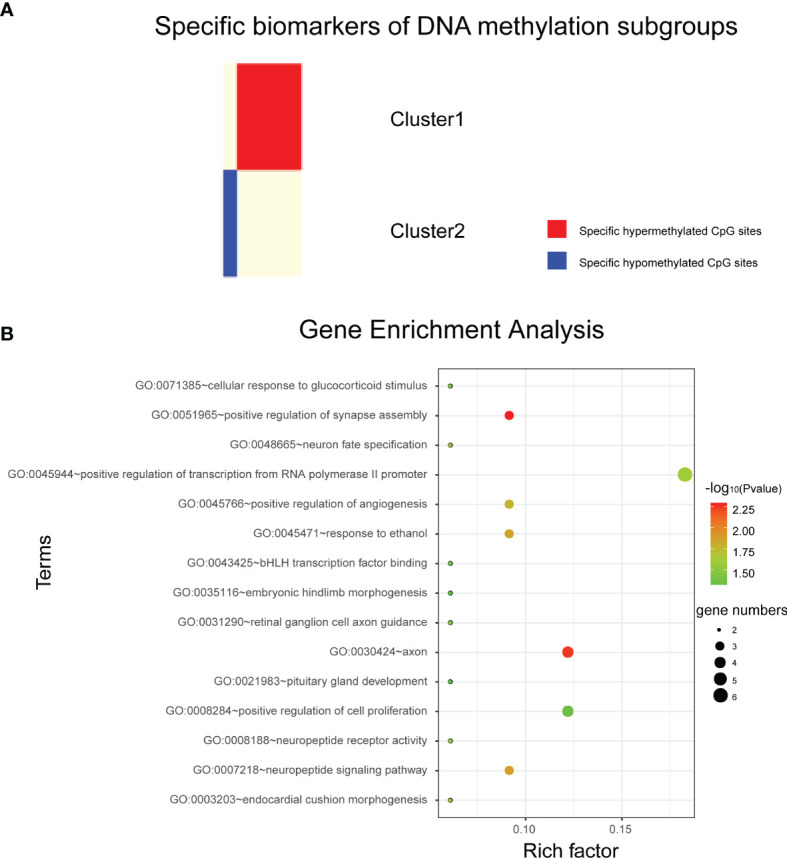
Analysis of subtype specific biomarkers. **(A)** Specific hyper/hypo methylated CpG sites for DNA methylation cluster1 and cluster2. **(B)** Gene enrichment analysis of genes corresponding to specific hypermethylated CpG sites in cluster2.

The QDMR software developed as a quantitative method described above was used in this study. 986 CpG loci across four DNA methylation subgroups (the classification features used in this study) were used as candidate features to screen for specific CpG markers in each subgroup. Since the methylation levels of these 986 CpG sites were used to distinguish the DNA methylation subgroups in this study, in each subgroup, these features should have similar methylation levels and there was very little variability between samples. Therefore, for each of the four DNA methylation subgroups, the average DNA methylation level of the 986 CpG sites in the samples was calculated to represent the DNA methylation pattern of that subgroup, and the 986× 4-dimensional result matrix was used as the input of QDMR. Finally, 52 specific hyper/hypomethylated CpG loci were identified, corresponding to 38 genes. They can be used as specific DNA methylation markers for different DNA methylation subgroups in bladder cancer, representing the unique DNA methylation patterns of that subgroup. The results showed that cluster1 and cluster2 specific CpG sites were found, and the number of CpG sites was 9 and 43, respectively. The specific sites of cluster1 screened out by our study are all hypomethylated, while the specific sites of cluster2 are all hypermethylated (**Figure 3A**).

Cluster1 specific CpG loci mapped 3 genes, and cluster2 specific CpG loci mapped 35 genes (**Supplementary Material 1**). Next, DAVID bioinformatics tool was employed to complete the Gene Ontology and KEGG pathway enrichment analysis on cluster2 specific genes to further explore the biological processes or pathways involved. Results as shown in **Figure 3B**, these genes are involved in biological processes such as positive transcriptional regulation of RNA polymerase II promoters. But we did not find pathways in which these genes were significantly enriched.

### Validation of DNA Methylation Molecular Subtypes and Subtype-Specific CpG Sites

To verify the robustness of the DNA methylation molecular subtypes of bladder cancer obtained in this study and the accuracy of the subtype specific CpG sites screened, we searched the GEO database and obtained a set of Illumina Infinium HumanMethylation450 Bead Chip DNA methylation profile data of urinary tumors (GSE52955), which included 25 patients with bladder cancer. Data from these 25 patients were used as a test set to verify the molecular subtypes and subtype-specific CpG sites obtained in this study.

**Figure 4:**
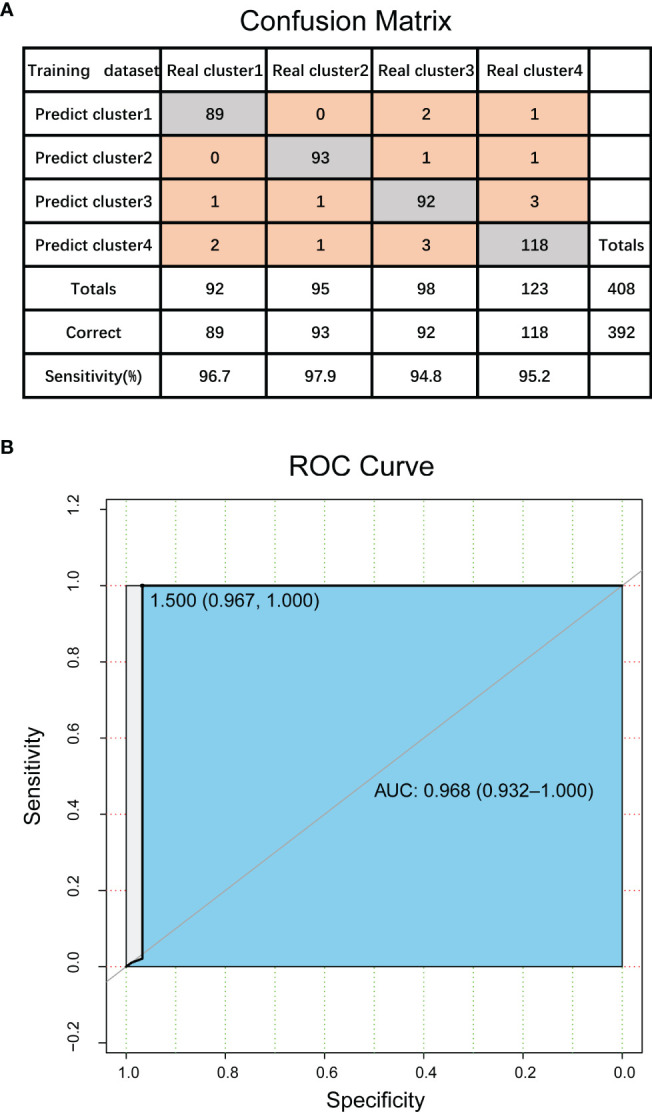
Validation of DNA methylation molecular subtypes and classification features in bladder cancer. **(A)** The confusion matrix of classification model. **(B)** ROC curve of classification model.

Firstly, we used 986 CpG loci previously screened as classification features, and constructed a support vector machine (SVM) classifier model using TCGA data set with classification labels (i.e., four DNA methylation molecular subtypes divided in this study). Here we conducted functional analysis of the genes corresponding to the 986 characteristic CpG loci, and found that they were enriched in regulation of transcription from RNA polymerase II promoter, cell differentiation, positive regulation of cell migration, cell-cell adhesion, signal transduction, negative regulation of cell proliferation, cAMP signaling pathway, vascular smooth muscle contraction and many other biological processes and pathways involved in cancer genesis and development. The model was verified using tenfold cross validation. The results showed that the classification accuracy of the model was 96%, sensitivity 96.1%, precision 96.1%, and area under ROC curve (AUC) reached 0.968 (**Figure 4**). This further proved the accuracy of the classification features screened in this study and the robustness of the DNA methylation molecular subtypes of bladder cancer.

Next, we input the test set obtained from GEO database into the constructed classifier model, which is used to predict the test set samples into the four DNA methylation molecular subtypes divided in this study. The 25 samples in the test set were predicted to be cluster1, cluster2, cluster3 and cluster4 with 3, 5, 11 and 6 samples respectively. Next, we tested the DNA methylation level of the subtype-specific CpG sites screened in this study in the test set. The results showed that 6 of the 9 CpG sites with cluster1-specific hypomethylation in the test dataset still had the lowest average methylation level in the four subtypes. The methylation level of the other three sites was not the lowest (close to the methylation level of cluster3), but significantly lower than that of the other two subtypes. In cluster2, all of the 43 CpG sites specific hypermethylated are still the CpG sites with the highest average methylation level among the four subtypes and significantly higher than the other three subtypes (**Supplementary Material 2**). This proves the accuracy and portability of the subtype-specific CpG sites screened in our study.

## Discussion

Cancer is a disease with high mortality rate and a serious threat to people’s health. Previous studies focused only on the effect of genetic sequence changes on cancer, or malignancy. Recently, a relationship between cancer and the level of DNA methylation has been found. TCGA database is a publicly available resource covering a wide variety of data types in a variety of cancers. The Infinium HumanMethylation450 BeadChip array dataset of bladder cancer contains a large number of samples that were downloaded from TCGA for our classification analysis. The large sample sizes allowed us to explore the molecular subtypes of bladder cancer more comprehensively.

Precision medicine in cancer treatment is based on the assumption that every patient has a unique variation of genetic alterations and should be treated accordingly. Thus, for personalized medicine to be effective, it is necessary to achieve a detailed classification of the cancer genome and epigenome. Many studies have suggested that epigenetic modifications (DNA methylation) play a pivotal role in early detection, and improved molecular classification, prognosis and adjuvant treatment of bladder cancer. These opinions suggested that the level of analysis could have important biological and clinical implications in the era of precision medicine (43, 44). Moreover, classifications based solely on the tissue of origin or pathological features have shown their limitations. To this end, we conducted this study to obtain molecular classifications of bladder cancer epigenomes based on DNA methylation.

In this study, DNA methylation and gene expression profile data of TCGA were integrated to screen differentially expressed genes and differentially DNA methylated CpG sites. CpG sites with significant negative correlation between methylation level and gene expression level were extracted as classification features. Then bladder cancer samples were classified according to the classification features, and four DNA methylation molecular subtypes were obtained. The prognostic difference analysis of these four subtypes showed that there were significant differences in the molecular level and prognostic status among these subtypes. Furthermore, subtype-specific biomarkers were identified using information entropy-based algorithm to represent the unique molecular characteristics of each subtype. These results suggest that there are significant differences in epigenetics and prognosis among subpopulations of patients with the same cancer, and clinicians may be able to develop personalized and timely treatment changes based on their prognostic status. However, the specific characteristic biomarkers for only two of the four DNA methylation molecular subtypes, namely cluster1 and cluster2, were identified. The specific characteristic biomarkers for cluster3 and cluster4 were not identified. This indicates that these two DNA methylated molecular subtypes are more similar at the molecular level, and their differentiation is not as obvious as the other two subtypes, which also brings certain limitations to this study. We hope that future studies can focus on further differentiation of these two subgroups.

In conclusion, our research identified four different molecular subgroups using the data of bladder tumors in TCGA. This is a more detailed explanation of the molecular heterogeneity of bladder cancer. The specific CpG sites and genes for particular subgroups can serve as biomarkers for personalized treatments. Changes in DNA methylation (hypo/hypermethylation) can be used as markers to diagnose particular subgroups, and clinicians can develop personalized treatments according to these prognoses. Additionally, our methods can also be used to study other tumors with high molecular heterogeneity.

## Data Availability Statement

Publicly available datasets were analyzed in this study. This data can be found here: All data analyzed in this study are from open data (freely available to anyone) at TCGA database and GEO database.

## Author Contributions

SZ and SH conceived and designed the experiments. SW, DX, YS, and SY conducted all the data processing work described in the section of methods and performed the analysis. SZ and BG prepared and edited the manuscript. XT and YJ checked and proofread the entire manuscript. All authors contributed to the article and approved the submitted version.

## Funding

This work was supported by National Natural Science Foundation of China (62002057, 62002087, 62172129); Fundamental Research Funds for the Central Universities (2572020BH02); Innovation and Entrepreneurship Training Program for Students of Northeast Forestry University (DC2020141).

## Conflict of Interest

The authors declare that the research was conducted in the absence of any commercial or financial relationships that could be construed as a potential conflict of interest.

## Publisher’s Note

All claims expressed in this article are solely those of the authors and do not necessarily represent those of their affiliated organizations, or those of the publisher, the editors and the reviewers. Any product that may be evaluated in this article, or claim that may be made by its manufacturer, is not guaranteed or endorsed by the publisher.

## Supplementary Material

Supplementary Material 1 Subtype-specific CpG sites and the corresponding genes.

Supplementary Material 2 Categorical sample specificity of each subtype-specific site.

## References

[1] Tang W Wan S Yang Z Teschendorff AE Zou Q. Tumor Origin Detection With Tissue-Specific miRNA and DNA Methylation Markers. Bioinformatics (2018) 34(3):398–406. doi: 10.1093/bioinformatics/btx622 29028927

[2] Jones PA Baylin SB. The Epigenomics of Cancer. Cell (2007) 128(4):683–92. doi: 10.1016/j.cell.2007.01.029 PMC3894624 17320506

[3] Li H Gong Y Liu Y Lin H Wang G. Detection of Transcription Factors Binding to Methylated DNA by Deep Recurrent Neural Network. Brief Bioinform (2022) 23(1):bbab533. doi: 10.1093/bib/bbab533 34962264

[4] Li E Zhang Y. DNA Methylation in Mammals. Cold Spring Harbor Perspect Biol (2014) 6(5):a019133. doi: 10.1101/cshperspect.a019133 PMC3996472 24789823

[5] Luo X Zhang T Zhai Y Wang F Zhang S Wang G. Effects of DNA Methylation on TFs in Human Embryonic Stem Cells. Front Genet (2021) 12:639461. doi: 10.3389/fgene.2021.639461 33708244 PMC7940757

[6] Mo F Luo Y Fan DA Zeng H Zhao YN Luo M. Integrated Analysis of mRNA-Seq and miRNA-Seq to Identify C-MYC, YAP1 and miR-3960 as Major Players in the Anticancer Effects of Caffeic Acid Phenethyl Ester in Human Small Cell Lung Cancer Cell Line. Curr Gene Ther (2020) 20(1):15–24. doi: 10.2174/1566523220666200523165159 32445454

[7] Zuo Y Song M Li H Chen X Cao P Zheng L. Analysis of the Epigenetic Signature of Cell Reprogramming by Computational DNA Methylation Profiles. Curr Bioinf (2020) 15(6):589–99. doi: 10.2174/1574893614666190919103752

[8] Tanaka E Uchida D Shiraha H Kato H Ohyama A Iwamuro M. Promising Gene Therapy Using an Adenovirus Vector Carrying REIC/Dkk-3 Gene for the Treatment of Biliary Cancer. Curr Gene Ther (2020) 20(1):64–70. doi: 10.2174/1566523220666200309125709 32148193

[9] Zhang S Zhang J Zhang Q Liang Y Du Y Wang G. Identification of Prognostic Biomarkers for Bladder Cancer Based on DNA Methylation Profile. Front Cell Dev Biol (2021) 9:817086. doi: 10.3389/fcell.2021.817086 35174173 PMC8841402

[10] Karsli-Ceppioglu S Dagdemir A Judes G Ngollo M Penault-Llorca F Pajon A. Epigenetic Mechanisms of Breast Cancer: An Update of the Current Knowledge. Epigenomics (2014) 6(6):651–64. doi: 10.2217/epi.14.59 25531258

[11] Jaenisch R Bird A. Epigenetic Regulation of Gene Expression: How the Genome Integrates Intrinsic and Environmental Signals. Nat Genet (2003) 33 Suppl:245–54. doi: 10.1038/ng1089 12610534

[12] Yalcin D Otu HH. An Unbiased Predictive Model to Detect DNA Methylation Propensity of CpG Islands in the Human Genome. Curr Bioinf (2021) 16(2):179–96. doi: 10.2174/1574893615999200724145835

[13] Sahu R Pattanayak SP. Strategic Developments & Future Perspective on Gene Therapy for Breast Cancer: Role of mTOR and Brk/PTK6 as Molecular Targets. Curr Gene Ther (2020) 20(4):237–58. doi: 10.2174/1566523220999200731002408 32807051

[14] Bariol C Suter C Cheong K Ku SL Meagher A Hawkins N. The Relationship Between Hypomethylation and CpG Island Methylation in Colorectal Neoplasia. Am J Pathol (2003) 162(4):1361–71. doi: 10.1016/S0002-9440(10)63932-6 PMC1851239 12651628

[15] Oster B Thorsen K Lamy P Wojdacz TK Hansen LL Birkenkamp-Demtroder K. Identification and Validation of Highly Frequent CpG Island Hypermethylation in Colorectal Adenomas and Carcinomas. Int J Cancer (2011) 129(12):2855–66. doi: 10.1002/ijc.25951 21400501

[16] Shen Z Zou Q. Basic Polar and Hydrophobic Properties are the Main Characteristics That Affect the Binding of Transcription Factors to Methylation Sites. Bioinformatics (2020) 36(15):4263–8. doi: 10.1093/bioinformatics/btaa492 32399547

[17] Cheng L Qi C Yang H Lu M Cai Y Fu T. Gutmgene: A Comprehensive Database for Target Genes of Gut Microbes and Microbial Metabolites. Nucleic Acids Res (2021) 50(D1):D795–800. doi: 10.1093/nar/gkab786 PMC8728193 34500458

[18] Beggs AD Jones A El-Bahrawy M Abulafi M Hodgson SV Tomlinson IP. Whole-Genome Methylation Analysis of Benign and Malignant Colorectal Tumours. J Pathol (2013) 229(5):697–704. doi: 10.1002/path.4132 23096130 PMC3619233

[19] Lao VV Grady WM. Epigenetics and Colorectal Cancer. Nat Rev Gastroenterol Hepatol (2011) 8(12):686–700. doi: 10.1038/nrgastro.2011.173 22009203 PMC3391545

[20] Luo Y Wong CJ Kaz AM Dzieciatkowski S Carter KT Morris SM. Differences in DNA Methylation Signatures Reveal Multiple Pathways of Progression From Adenoma to Colorectal Cancer. Gastroenterology (2014) 147(2):418–429.e418. doi: 10.1053/j.gastro.2014.04.039 24793120 PMC4107146

[21] Mohammed M Mwambi H Omolo B. Colorectal Cancer Classification and Survival Analysis Based on an Integrated RNA and DNA Molecular Signature. Curr Bioinf (2021) 16(4):583–600. doi: 10.2174/1574893615999200711170445

[22] Gao J Zhang L Yu G Qu G Li Y Yang X. Model With the GBDT for Colorectal Adenoma Risk Diagnosis. Curr Bioinf (2020) 15(9):971–9. doi: 10.2174/1574893614666191120142005

[23] Liu Q Wan J Wang G. A Survey on Computational Methods in Discovering Protein Inhibitors of SARS-CoV-2. Brief Bioinform (2022) 23(1):bbab416. doi: 10.1093/bib/bbab416 34623382 PMC8524468

[24] Li Y Qiao G Wang K Wang G. Drug-Target Interaction Predication via Multi-Channel Graph Neural Networks. Brief Bioinform (2022) 23(1):bbab346. doi: 10.1093/bib/bbab346 34661237

[25] Li Y Wang K Wang G. Evaluating Disease Similarity Based on Gene Network Reconstruction and Representation. Bioinformatics (2021) btab252. doi: 10.1093/bioinformatics/btab252 33978702

[26] Gerlinger M Catto JW Orntoft TF Real FX Zwarthoff EC Swanton C. Intratumour Heterogeneity in Urologic Cancers: From Molecular Evidence to Clinical Implications. Eur Urol (2015) 67(4):729–37. doi: 10.1016/j.eururo.2014.04.014 24836153

[27] Yuk HD Kim JK Jeong CW Kwak C Kim HH Ku JH. Differences in Pathologic Results of Repeat Transurethral Resection of Bladder Tumor (TURBT) According to Institution Performing the Initial TURBT: Comparative Analyses Between Referred and Nonreferred Group. BioMed Res Int (2018) 2018:9432606. doi: 10.1155/2018/9432606 30271788 PMC6146742

[28] Kawakami T Shiina H Igawa M Deguchi M Nakajima K Ogishima T. Inactivation of the Hmsh3 Mismatch Repair Gene in Bladder Cancer. Biochem Biophys Res Commun (2004) 325(3):934–42. doi: 10.1016/j.bbrc.2004.10.114 15541380

[29] Liu Z Sun T Zhang Z Bi J Kong C. An 18-Gene Signature Based on Glucose Metabolism and DNA Methylation Improves Prognostic Prediction for Urinary Bladder Cancer. Genomics (2021) 113(1 Pt 2):896–907. doi: 10.1016/j.ygeno.2020.10.022 33096258

[30] Burki TK. High Genetic Heterogeneity in Some Breast Cancer Tumours. Lancet Oncol (2015) 16(15):e529. doi: 10.1016/S1470-2045(15)00359-9 26411399

[31] Chen Y Zhou J Xu Y Li Z Wen X Yao L. BRCA1 Promoter Methylation Associated With Poor Survival in Chinese Patients With Sporadic Breast Cancer. Cancer Sci (2009) 100(9):1663–7. doi: 10.1111/j.1349-7006.2009.01225.x PMC11158407 19522853

[32] Tan TZ Rouanne M Tan KT Huang RY Thiery JP. Molecular Subtypes of Urothelial Bladder Cancer: Results From a Meta-Cohort Analysis of 2411 Tumors. Eur Urol (2019) 75(3):423–32. doi: 10.1016/j.eururo.2018.08.027 30213523

[33] Lindskrog SV Prip F Lamy P Taber A Groeneveld CS Birkenkamp-Demtroder K. An Integrated Multi-Omics Analysis Identifies Prognostic Molecular Subtypes of non-Muscle-Invasive Bladder Cancer. Nat Commun (2021) 12(1):2301. doi: 10.1038/s41467-021-22465-w 33863885 PMC8052448

[34] Robertson AG Kim J Al-Ahmadie H Bellmunt J Guo G Cherniack AD. Comprehensive Molecular Characterization of Muscle-Invasive Bladder Cancer. Cell (2017) 171(3):540–556.e525. doi: 10.1016/j.cell.2017.09.007 28988769 PMC5687509

[35] Ye F Liang Y Hu J Hu Y Liu Y Cheng Z. DNA Methylation Modification Map to Predict Tumor Molecular Subtypes and Efficacy of Immunotherapy in Bladder Cancer. Front Cell Dev Biol (2021) 9:760369. doi: 10.3389/fcell.2021.760369 34926451 PMC8678484

[36] Wilhelm-Benartzi CS Koestler DC Houseman EA Christensen BC Wiencke JK Schned AR. DNA Methylation Profiles Delineate Etiologic Heterogeneity and Clinically Important Subgroups of Bladder Cancer. Carcinogenesis (2010) 31(11):1972–6. doi: 10.1093/carcin/bgq178 PMC2966555 20802236

[37] Cancer Genome Atlas Research N Weinstein JN Collisson EA Mills GB Shaw KR Ozenberger BA. The Cancer Genome Atlas Pan-Cancer Analysis Project. Nat Genet (2013) 45(10):1113–20. doi: 10.1038/ng.2764 PMC3919969 24071849

[38] Wu H Zhang Y. Reversing DNA Methylation: Mechanisms, Genomics, and Biological Functions. Cell (2014) 156(1-2):45–68. doi: 10.1016/j.cell.2013.12.019 24439369 PMC3938284

[39] Wilkerson MD Hayes DN. ConsensusClusterPlus: A Class Discovery Tool With Confidence Assessments and Item Tracking. Bioinformatics (2010) 26(12):1572–3. doi: 10.1093/bioinformatics/btq170 PMC2881355 20427518

[40] Zhang Y Liu H Lv J Xiao X Zhu J Liu X. QDMR: A Quantitative Method for Identification of Differentially Methylated Regions by Entropy. Nucleic Acids Res (2011) 39(9):e58. doi: 10.1093/nar/gkr053 21306990 PMC3089487

[41] Huang D Sherman BT Lempicki RA. Bioinformatics Enrichment Tools: Paths Toward the Comprehensive Functional Analysis of Large Gene Lists. Nucleic Acids Res (2009) 37(1):1–13. doi: 10.1093/nar/gkn923 19033363 PMC2615629

[42] Huang da W Sherman BT Lempicki RA. Systematic and Integrative Analysis of Large Gene Lists Using DAVID Bioinformatics Resources. Nat Protoc (2009) 4(1):44–57. doi: 10.1038/nprot.2008.211 19131956

[43] Pasculli B Barbano R Parrella P. Epigenetics of Breast Cancer: Biology and Clinical Implication in the Era of Precision Medicine. Semin Cancer Biol (2018) 51:22–35. doi: 10.1016/j.semcancer.2018.01.007 29339244

[44] Hu WL Zhou XH. Identification of Prognostic Signature in Cancer Based on DNA Methylation Interaction Network. BMC Med Genomics (2017) 10(Suppl 4):63. doi: 10.1186/s12920-017-0307-9 29322932 PMC5763425

